# Plant–environment microscopy tracks interactions of *Bacillus subtilis* with plant roots across the entire rhizosphere

**DOI:** 10.1073/pnas.2109176118

**Published:** 2021-11-24

**Authors:** Yangminghao Liu, Daniel Patko, Ilonka Engelhardt, Timothy S. George, Nicola R. Stanley-Wall, Vincent Ladmiral, Bruno Ameduri, Tim J. Daniell, Nicola Holden, Michael P. MacDonald, Lionel X. Dupuy

**Affiliations:** ^a^School of Science and Engineering, University of Dundee, Dundee DD1 4HN, United Kingdom;; ^b^Ecological Sciences, The James Hutton Institute, Dundee DD2 5DA, United Kingdom;; ^c^Department of Conservation of Natural Resources, Neiker, Derio 48160, Spain;; ^d^School of Life Sciences, University of Dundee, Dundee DD1 5EH, United Kingdom;; ^e^Institut Charles Gerhardt de Montpellier, Université de Montpellier, CNRS, ENSCM, Montpellier 34090, France;; ^f^Plants, Photosynthesis and Soil, School of Biosciences, The University of Sheffield, Sheffield S10 2TN, United Kingdom;; ^g^Northern Faculty, Scotland’s Rural College, Aberdeen AB21 9YA, United Kingdom;; ^h^Ikerbasque, Basque Foundation for Science, Bilbao 48009, Spain

**Keywords:** environmental imaging, root–microbe interactions, rhizosphere

## Abstract

Our understanding of plant–microbe interactions in soil is limited by the difficulty of observing processes at the microscopic scale throughout plants’ large volume of influence. Here, we present the development of three-dimensional live microscopy for resolving plant–microbe interactions across the environment of an entire seedling growing in a transparent soil in tailor-made mesocosms, maintaining physical conditions for the culture of both plants and microorganisms. A tailor-made, dual-illumination light sheet system acquired photons scattered from the plant while fluorescence emissions were simultaneously captured from transparent soil particles and labeled microorganisms, allowing the generation of quantitative data on samples ∼3,600 mm^3^ in size, with as good as 5 µm resolution at a rate of up to one scan every 30 min. The system tracked the movement of *Bacillus subtilis* populations in the rhizosphere of lettuce plants in real time, revealing previously unseen patterns of activity. Motile bacteria favored small pore spaces over the surface of soil particles, colonizing the root in a pulsatile manner. Migrations appeared to be directed toward the root cap, the point of “first contact,” before the subsequent colonization of mature epidermis cells. Our findings show that microscopes dedicated to live environmental studies present an invaluable tool to understand plant–microbe interactions.

## Significance

The lack of suitable approaches for studying root–microbe interactions, live and in situ, has severely limited our ability to understand the rhizosphere. In this study, we overcome this major limitation with an imaging system that combines transparent soils with cutting edge light sheet microscopy. The study revealed that the root cap is a point of first contact for microbes before establishment and reveals how the pore structure influences the patterns of interactions between the microbe and the plant. With the combined use of light sheet microscopy and transparent soils, we shed light on previously unseen interaction phenomena and accelerate the understanding of how rhizospheres are formed.

The ability of plants and microorganisms to cooperate to capture soil resources underpins life in terrestrial ecosystems. In modern crop production systems, in which these natural plant–microbe interactions have largely been replaced by artificial fertilizer input, it is thought that crop varieties may have lost the ability to maintain a diverse microbiome (1), and as a consequence, the sustainability of the system has declined. Consequently, understanding of plant–microbe interactions has become a major focus of research. Technological development has greatly expanded the knowledge of the microbial composition of soil: Metabolomics detail the chemical composition of organic material deposited by the root and high-throughput sequencing now describes the huge complexity of microbial communities associated with them (2). Soil habitats, however, are incredibly dynamic and structurally complex. The behavior of the microbes inhabiting the inner structures of soil are equally complex, and to date, current approaches have failed to provide mechanistic understanding of soil microbial dynamics (3).

Since the discovery of microorganisms, microscopy has constantly improved, and modern microscopes are now able to solve problems of considerable complexity (4, 5). However, live microscopy of plants within the biotic and abiotic environment remains complex and rarely achieved. Processes within the opaque world hidden within the soil structure are particularly difficult to monitor. Current microscopy methods applicable to soil are either destructive (6, 7), operate with samples of extremely limited volume and area (8), or oversimplify the role of the physical and chemical structure of the soil material (9). Maintaining a viable, undisturbed biological system is also a challenging condition to meet in the laboratory because processes occur both below and above ground, with different controls required for light, temperature, water, and mineral content (10).

The aim of this study was to build an “environmental microscope,” which we define as a live-sample imaging platform dedicated to the observation of physical and biological interactions that are relevant to the understanding of processes at environmental or system levels. The platform we propose exploits recent advances in transparent soils, mesofluidics, and light sheet imaging. It acquires both fluorescence emissions and elastically scattered photons across the entire spatial domain surrounding a plant root, simultaneously combining all necessary controls for light, temperature, and water content within the mesocosm. This study reveals previously unobserved phenomena of how bacteria colonize the rhizosphere, the region of soil surrounding plant root, and demonstrate the potential of the method to fill important knowledge gaps in environmental biology.

## Results

### Light Sheet Imaging for Whole-Plant Environment Microscopy.

**Fig. 1.**
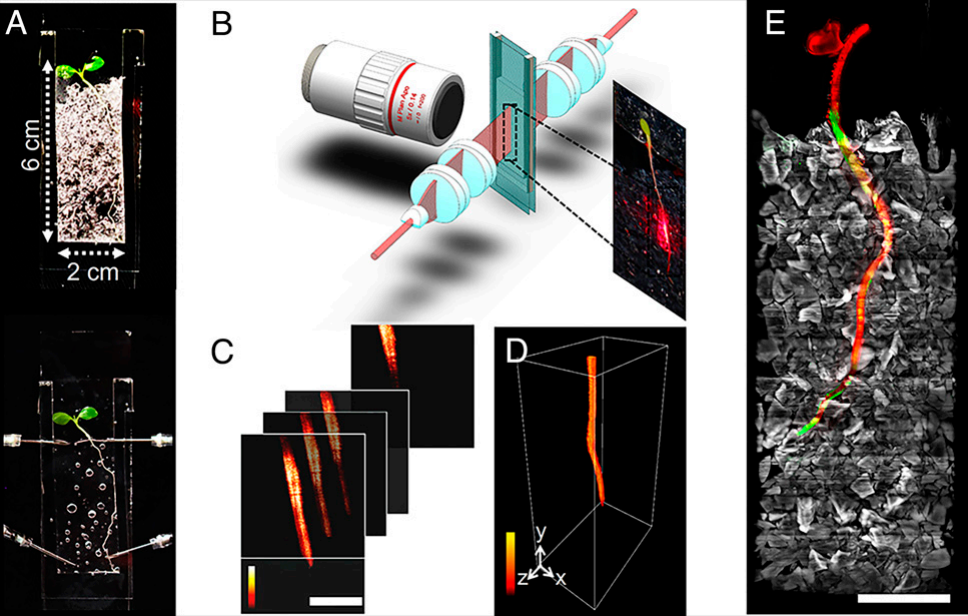
Live microscopy of the whole-plant environment. (*A*, *Top*) Samples consisted of mesocosms filled with transparent soil and coinoculated with lettuce plants and *B. subtilis*. (*Bottom*) To perform imaging, the sample is saturated in refractive, index-matching liquid. (*B*) Light sheet microscopy (not drawn to scale) consists of a long focus homogenous light sheet generated using a Powell and two cylindrical lenses. The light sheet produces fluorescence and scattering signals captured by a long working distance objective. The mesocosm is immersed in refractive index-matched solution and is positioned and translated at a 45° angle within the light sheet and detection arm. (*C*) Image data are acquired by translating the sample first in the horizontal plane, then vertically. (Scale bar, 2 mm.) (*D*) A complete volume dataset is stitched from a series of horizontal scans. (*E*) The microscope captured volume data of up to 3 × 60 × 20 mm^3^ and tracked the growth of entire seedlings (scattering, red), transparent soil particles (sulforhodamine B fluorescence, grey), and bacterial concentration (GFP-tagged microorganisms *B. subtilis*, green). (Scale bar, 5 mm.)

Observations were made from lettuce seedlings, a tractable, important crop plant for mesocosm studies, and *Bacillus subtilis*, a well-characterized rhizobacterium with potential for biocontrol applications. Custom-made chambers were assembled from glass slides and silicon parts to seal 4,290 mm^3^ of transparent soil, water, nutrients, and atmosphere (in a cuboid of 22 × 65 × 3 mm^3^). The model system studied, therefore, was the entire environment supporting the lives of both plants and microbes (Fig. 1*A* and *SI Appendix*, Supplementary Texts 1–3).

Index matching of the soil was achieved using Percoll, a nontoxic colloid suspension that did not negatively impact the growth and mobility of *B. subtilis* (*SI Appendix*, Supplementary Text 3). Colonization of lettuce roots with *B. subtilis* did not significantly impact elongation rate (*SI Appendix*, Supplementary Text 3).

Acquiring biological signals from such volumes necessitates instruments that combine dedicated microscopy with adequate control hardware and software. In this study, we showed that light sheet fluorescence microscopy (LSFM) meets requirements for scale, throughput, and integration with live mesocosm (11). The light sheet sectioned the sample optically with laser illumination optics, and the camera sensor captured both the fluorescence emissions and scattered photons perpendicular to the plane of the light sheet (Fig. 1*B*). This was preferable to the use of condenser lenses and objectives with high numerical aperture (NA) for illumination and image capture because a high-NA lens creates a shallow depth of focus (12) and limited field of view, both of which are incompatible with the imaging of large samples.

To achieve adequate throughput, and to limit the need for the stitching of multiple views, large field of view objectives (5× or 2× objectives with a field of view 2.4 or 6 mm, respectively) were combined with a light sheet a centimeter in width and height. The light sheet was generated with Powell lenses (13) and a series of cylindrical lenses that focused the sheet to the focal plane of the imaging objective with a thickness of 50 µm (from theoretical limit of 47 µm), a height of more than 5 mm, and a Rayleigh range (depth of focus) of at least 6 mm (*SI Appendix*, Supplementary Texts 4 and 5).

**Fig. 2.**
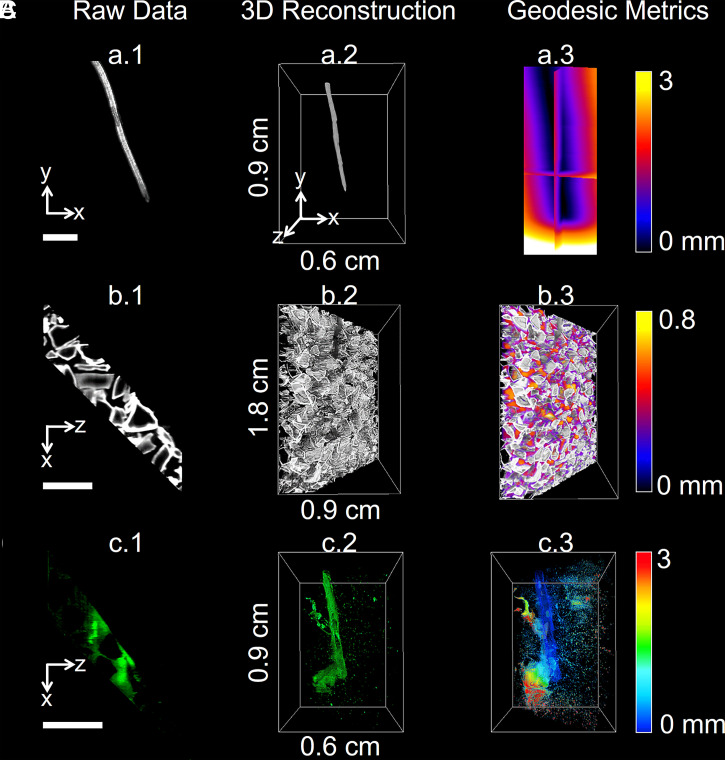
Quantification of root–soil–bacteria interactions. Image data from lettuce root (*A*), transparent soil particles (*B*), and GFP-labeled *B. subtilis* (*C*). Processing of the data follows three steps. (*Left*) Raw data are acquired from the microscope. (*Middle*) Cross-sections are assembled into volume data through stitching and stacking. (*Right*) Image processing is subsequently performed to quantify temporal and spatial patterns of biological activity in the pore space. The metrics obtained from the data include distance from the root surface (*A*), pore size (*B*), and bacterial cell density (*C*). (Scale bar, 2 mm.)

Data from the entire mesocosm volume was successfully reconstructed (Fig. 1 *C* and *D*) from custom-made software that aligned the overlapping scans, corrected for artifacts from the imaging system, and produced a unique volume image containing signals from root, bacteria, and soil particles (Movie S1 and *SI Appendix*, Supplementary Texts 6 and 7). The data generated by the microscope (Fig. 1*E*) was suitable for the quantification of biological features by image analysis. The fluorescence from the bacteria was calibrated to predict cell density. Segmentation of the fluorescence signal detected from particles reported on how bacteria utilized the soil microstructure while the scattering signal from the root allowed the size and geometry of the root to be measured to accurately position bacterial population during growth, migration, and colonization on the root surface (Fig. 2 and Movies S1 and S2).

### Environmental Microscopy Resolves Bacterial Dynamics in the Pore Space.

The microcosms were set up in a controlled manner so that inoculated bacteria originated from a single source point, allowing their movement to be assessed (*SI Appendix*, Supplementary Text 1). In natural systems, bacteria are likely to encounter roots from a wide range of sources, and here, we simulate the situation in which movement toward the developing root is required prior to colonization. We observed movements of GFP producing *B. subtilis* strain NCIB 3610 within the soil volume (∼2/3 of the volume of the microcosm), as well as interactions with the surface of growing lettuce roots. Quantification was performed every hour over 23 h. The data acquired during those experiments were used to map the bacterial cell density in relation to the distance from the root surface (Fig. 2 *C, Right*), the distance along the root, and the size of soil pores. All of the dataset was reconstructed and assessed visually using the three-dimensional visualization pipeline described (*SI Appendix*, Supplementary Texts 6 and 7 and Movies S3–S5). Plant roots exhibited large variations in the total abundance of microbial cells, possibly due to changes in exudation pattern or root size (*SI Appendix*, Supplementary Text 8). To characterize colonization pattern independently of the magnitude of the colonization, the cell density was normalized (*SI Appendix*, Supplementary Text 8 and Eq. 2), and the spatial distribution of bacterial cells was analyzed as a function of distance from the root surface, position along the root and in the pore space. Analysis of root growth in the absence of bacteria was performed on four control samples (*SI Appendix*, Supplementary Text 3).

**Fig. 3.**
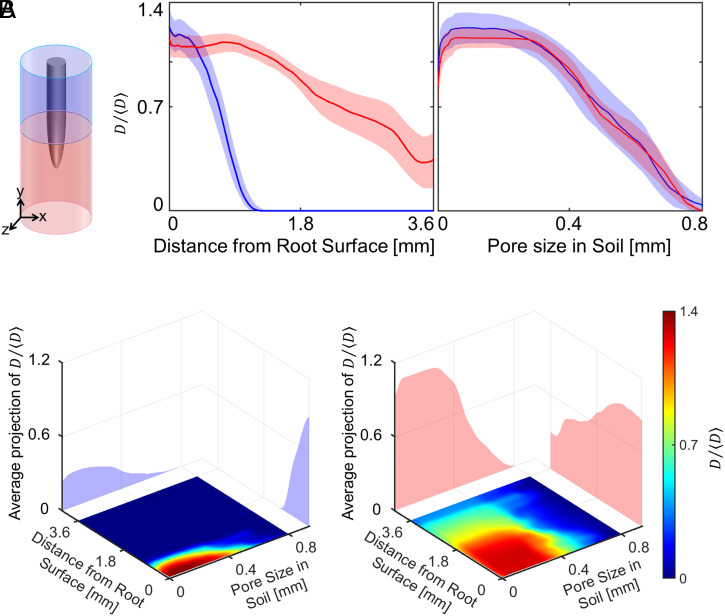
Utilization of the pore space by *B*. *subtilis* during colonization across the whole dataset. (*A*) The distribution of bacterial cell density varies as a function of the distance from the root surface. When bacteria are surrounding the basal region of the root (blue), they are present primarily in a radius of 1 mm around the root. When surrounding or in front of the root tip (red), bacteria are found within a radius around the root that is larger than 3 mm. There is little difference in the distribution of bacteria in the pore space. Data are shown as mean ± SE, *n* = 6. (*B*) The occupation of the pore space varies with the distance from the root. Bacteria tend to occupy the pore space more evenly when further away from the root; however, the effect is more visible when bacteria are surrounding the basal region of the root (*Left*) than near the root tip (*Right*). Data for the individual replicate are supplied in *SI Appendix*, Supplementary Text 8.

Overall, bacterial cell density estimated from pixel intensity (*SI Appendix*, Supplementary Text 6) was significantly greater when closer to the root surface. The phenomenon was particularly visible in the soil surrounding the base of the root (Fig. 3 *A*, *Left*), where bacterial cells density declines as a function of the distance from the root surface (Movie S6). By contrast, bacteria surrounding the root tip were observed within a radius of more than 3 mm from the root surface. Although the presence of bacteria was detected in all pore sizes, bacteria preferentially occupied smaller pores (<400 μm) of the soil (Fig. 3 *A*, *Right*).

We observed a weak relationship between the pore size occupied by bacterial cells and the distance from the root surface. Plots of joint bacterial cell density distribution (Fig. 3*B*) showed that bacteria closer to the root surface occupied the smaller pore spaced (<400 μm) in both the apical (Fig. 3 *B*, *Left*) and basal region (Fig. 3 *B*, *Right*) of the soil. On the contrary, bacteria from the bulk soil occupied the pore spaces more evenly.

### Bacteria Form Hotspots and Colonize the Rhizosphere in Pulses.

**Fig. 4.**
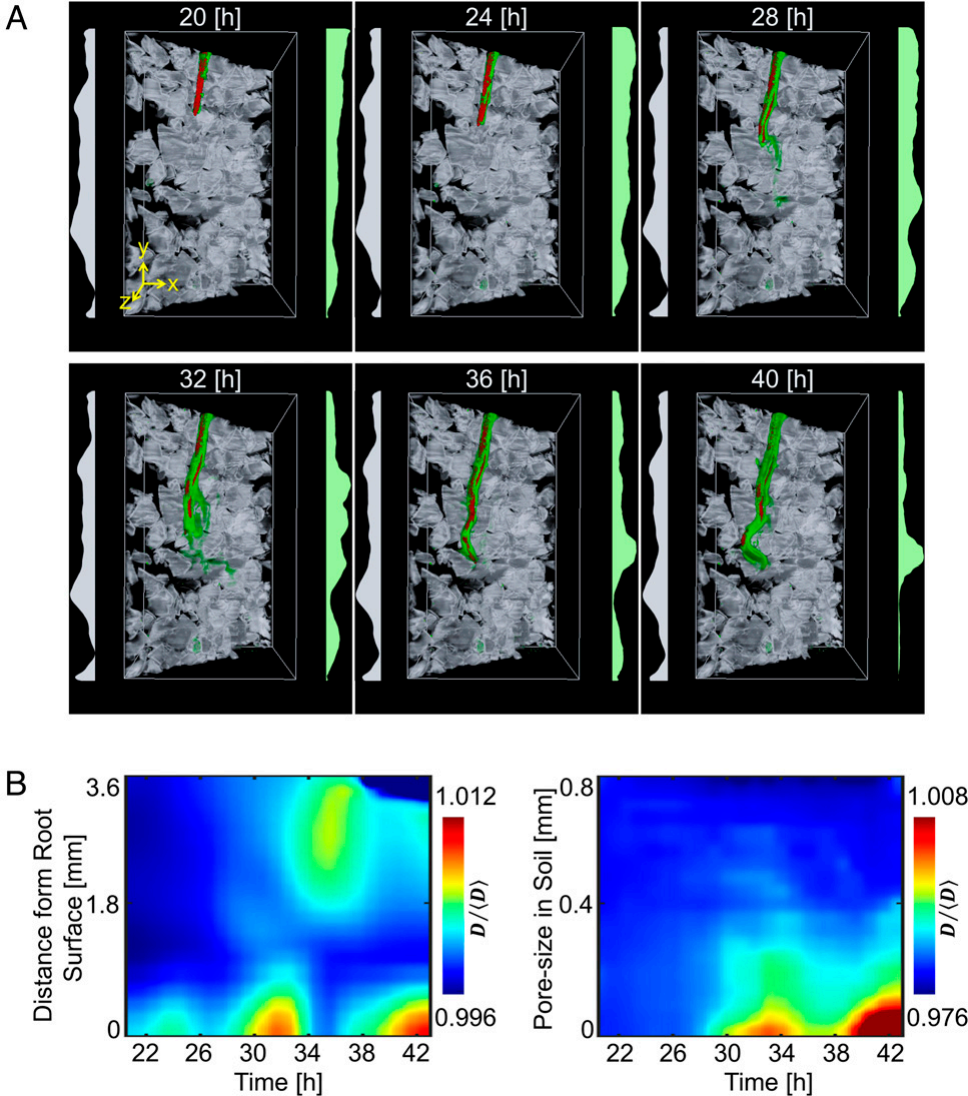
Time lapse data of the distribution of *B*. *subtilis* in transparent soil surrounding one lettuce root observed for 23 h after inoculation. (*A*) Three-dimensional visualization of bacterial cell density reveals highly dynamic patterns due to interactions with the microstructure of soil. Plots on the left (grey) show the average pore size, and plots on the right (green) show the average bacterial density. (*B*, *Left*) During colonization, bacterial cell density increased in pulses (∼4 h apart), forming patches of bacteria close to the root surface. (*Right*) The increase in bacterial cell density occurs primarily in the smaller pore space.

Unlike growth in liquid culture, soil provides a physical support for bacterial attachment but limits movement and confines cell activity to the pore microenvironment. To better understand how the pore space segregates the activity of *B. subtilis* during root colonization, a detailed analysis of the population dynamics was needed. Time-lapse data (over 23 h) revealed increased bacterial cell density forming in specific regions of the soil, forming “hotspots” close to the root or in the soil surrounding the root tip. Increased bacterial cell density was also observed on the root surface via attachment and/or biofilm formation (Fig. 4*A*). The location of bacterial hotspots varied significantly with time. Changes occurred more frequently near the root tip, and hotspots appeared to stabilize on mature parts of the tissue, indicating the attachment and formation of biofilms.

Changes in bacterial cell density over time indicated that rhizosphere colonization was pulsatile (Fig. 4*B*). Hotspots of bacteria appeared ∼25 h after inoculation in the smaller pore sizes (Fig. 4 *B*, *Right*) and was maintained for typically 2 to 4 h following their appearance (Fig. 4 *B*, *Left*). Hotspots were observed at distances of more than 3 mm from the root surface, although the distance appeared to diminish during the course of the colonization.

### Early Interaction with the Root Cap May Precede Colonization of the Root Surface.

**Fig. 5.**
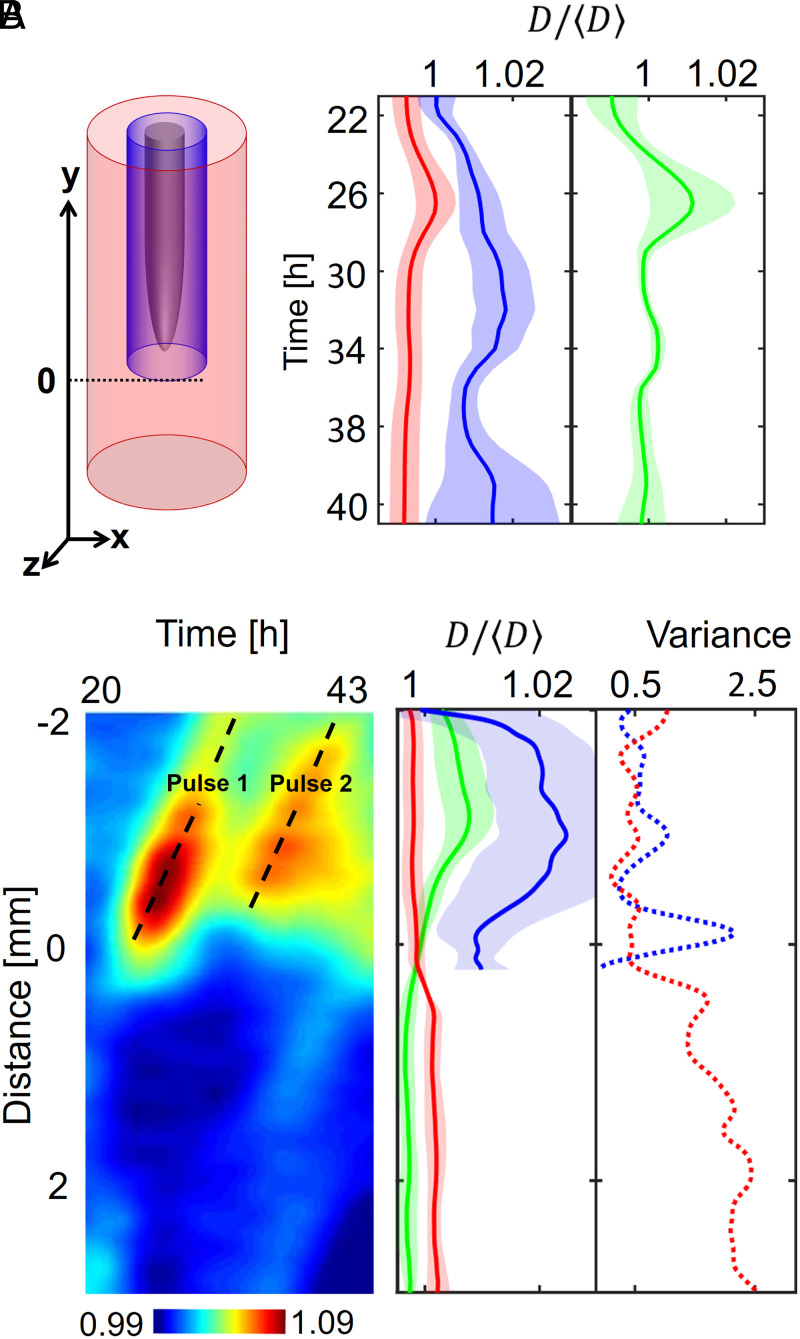
Dynamics of the colonization of lettuce roots by *B. subtilis* across the whole dataset. (*A*) The colonization is marked by an increase in bacterial cell density in the soil further away from the root (red) within the first 26 h following inoculation, after which a maximum is reached between 30 and 34 h following inoculation. Bacterial cell density increases more persistently closer to the root surface (blue) until 30 to 34 h following inoculation and does not really reach a steady state in the analyzed time frame. Even though the bacteria total quantity seemed to reach a maximum after 26 h (green), variations in cell density along the root persisted. This indicates that migration may play a role in the later stages of the colonization of the root. (*B*) Intense activity at the root tip may precede colonization near or on the root surface. (*Left*) Example of the diagram of the colonization kinematics shows how cell density changes both with time and as a function of the position along one root. The diagram shows that densification of bacterial cell population is discrete (here, two pulses 7 h apart are recorded at ∼1 mm from the tip) and likely results from the attachment of bacteria on nongrowing tissue, since diagonal patterns indicate the constant increase in the distance from the root tip. (*Middle*) Overall, the distribution of bacterial cell density along the root (solid blue line) confirms that bacterial cell density concentrates in the basal region of the root (>1 mm from the root tip). (*Right*) On the contrary, the most intense temporal variations in bacterial cell density are observed near the root tip (dashed blue line). The activity of bacterial cells in the bulk soil (red) confirmed bacterial activity at the tip of the root is enhanced, with both the density in the bulk soil (solid red line) and the variance (dashed red line) showing a maximum in the region near the root tip. Data shown as mean ± SE, *n* = 6.

Large differences in bacterial presence were observed in the bulk soil and in the rhizosphere (Fig. 5*A* and Movies S6–S8). For the purpose of this study, we have defined the rhizosphere as the soil volume surrounding the root up to a distance of 0.2 mm from the rhizoplane. The increase in mean bacterial cell density was first observed in the bulk soil 26 h after inoculation (Fig. 5*A*, red). Following a peak in bacterial cell density, the population of bacteria in the bulk soil subsequently reduced and reached a steady state. The increase in bacterial cell density in the rhizosphere (soil volume around the root up to 0.2 mm from the root surface) was more gradual and reached a peak 20 to 34 h after inoculation (Fig. 5*A*, blue). Fluctuations were observed following the peak of bacterial activity, but bacterial cell density remained high until termination of the experiment. The overall quantity of bacteria, calculated across the soil volume in the system, did not increase after reaching the peak concentration in the bulk soil (Fig. 5*A*, green). This indicates that subsequent changes in bacterial cell density may largely be induced by bacterial movements through soil and along the root.

To understand whether hotspots of bacteria move along the root, diagrams of the colonization kinematics of individual roots were obtained (Fig. 5 *B*, *Left*). Bacterial hotspots (high–bacterial cell density) appeared as diagonal stripes in the space–time domain, which showed that bacterial hotspots were mostly immobile. Hotspots are formed, therefore, from bacteria converging toward sites of attachment on the root surface or on surrounding soil particles.

The system did not allow the tracking of individual cells across the soil volume. To understand the movements of bacterial cells preceding attachment, we studied the variance in bacterial cell density (*SI Appendix*, Supplementary Text 8 and Eq. 4) along the root. We could establish a relationship between the variance of the cell density and the bacterial cell density itself along the root. As described earlier, the bacterial cell density was found to be larger in the basal and more mature part of the root at a distance starting ∼1 mm from the root tip (Fig. 5*B*, solid blue line). In contrast, bacteria from the bulk soil concentrated in the opposite direction at a distance larger than 500 µm from the root tip. The variance of bacterial cell density revealed the sites of high cell mobility and identified regions of soil where bacterial mobility was most intense. Large variances were recorded close to the root tip (Fig. 5*B*, dashed blue) and in the soil in front of the root tip (Fig. 5*B*, dashed red). This indicates the root cap may be a point of “first contact” for bacteria, with attachment and colonization occurring at a later stage and on the elongation zone of the root.

## Discussion

Performing live microscopy on plant–soil–atmosphere systems is challenging because of the necessity to maintain suitable conditions to grow plants and microorganisms, both in soil and within the confined space of a microscope. Many of these constraints are inherent to the observation and imaging of living organisms (14, 15). As a result of these challenges, and despite the importance of imaging organisms in situ, attempts to observe environmental interactions using live microscopy in solid, structured media have remained extremely limited. To date, the vast majority of the understanding of root–microbe dynamics is based on experiments performed in hydroponic or agar cultures (9, 16–18), which bear little resemblance to reality, and by extraction in which scales cannot be assessed properly.

At the other end of the spectrum, environmental or ecophysiological studies are limited by technologies for direct in situ observation of biological processes. Recent developments in neutron and X-ray tomography (19, 20) have revealed the complex nature of the interactions between root systems and soils and the diversity of their responses to each other (21). However, the techniques available are slow, and detection of microorganisms is limited or nonexistent. Other techniques such as laser ablation tomography (22) promise fast quantification of biological structures of roots within undisturbed soil cores but in a destructive manner.

Here, we have developed microscopy technologies that help bridge the gap between environmental and biological sciences. The technique combines the use of a transparent soil that allows the control of water, light, and nutrient supply within mesocosms and the circulation of refractive, index-matching liquid to image an entire plant and its surrounding environment. Using the system, root–soil–bacteria interactions were observed in situ, and we have gathered evidence that soil microstructure affects bacterial behavior (Movies S6–S8). The core of the system is a combined fluorescence and scattering light sheet microscope. The microscope illuminates a single sheet or slice of the sample while, perpendicular to the illumination plane, a camera captures both fluorescence and scattered photons from the sample (11, 23–25).

Light sheet microscopy has seen a growing number of applications in microbiology and plant sciences. For instance, the technique tracked growing root meristems for several days (26), captured rapid events such as calcium oscillation in root hairs (27), and monitored microbial communities during the formation of biofilm (28, 29) while recently super-resolution has been achieved (30). Current systems are tailored to image small-scale processes and are limited in their field of view or by the size of samples that can be imaged. There have been only limited attempts at utilizing light sheet microscopy to image roots through heterogeneous substrates and recover fluorescent signals from it (24). Attempts at using microfluidic systems to increase throughput are particularly promising to overcome the limitation to field of view while controlling growth conditions (9, 31), but growth conditions remain remote to those observed in natural soils.

Here, we have demonstrated an approach that overcomes limitations to imaging live roots and their surrounding biotic environment. The environmental microscope enables the scanning of the whole-root system of a juvenile plant and the capture of the microbial activity around roots for several days, enabling capture of the dynamics of the interactions. By collecting scattered light, alongside the fluorescence emissions from the sample, it is possible to image agronomically important crops without the requirement for genetic transformation or complex live staining steps. Hence, the system enhances the number of signals collected and could be used in a host of other research questions, combining, for example, fluorescent reporters in plants (32), mixtures of bacterial species (9), or using dynamic light scattering to track nematodes or fungal cells (33).

Results showed that around root tips, where bacteria were more mobile, the occupation of the pore space was evenly distributed. However, when bacteria established on or in the vicinity of the root, high–bacterial cell densities were observed primarily in smaller pores, confirming experiments made on fixed samples (34, 35). Even though bacterial motility in soil is essential for successful colonization of the rhizosphere (36), the complex heterogeneity of soil is known to limit microbial mobility (37) because of the effect of contact with surfaces and the confinement by obstacles (38). We found that a complex matrix such as soil may cause bacteria to grow and appear in pulses, targeting root surfaces as a group and forming patches on the root surface. The study gave insight into how soil structure modulates the dynamics of bacteria around growing roots.

Observations also hinted at the possibility that complex movements of cells occur before attachment on and colonization of the root surface. As described earlier, *B. subtilis* move chemotactically toward root surfaces (39), with high numbers of active bacteria commonly observed around the root tip (40), while biofilms are usually formed in the root elongation zone (9). In our system, attachment of the bacteria was observed 1 mm from the root apex, in a region that corresponds well to the end of the elongation zone. However, the increased sensitivity and ability to monitor microbial dynamics allowed us to identify other regions of importance to the microbial colonization process. A peak in bacterial mobility was consistently observed at the root apex. This peak of activity differed from the accumulation of cells in the root tip seen with *B. subtilis*, as well as in other bacterial species (6), because high mobility was associated with low–bacterial cell density. This activity could be linked to attraction and interaction with specific cell types [e.g., border cells and mucilage released by the root cap (41)].

This study demonstrates the ability of live microscopy to observe plant and microorganisms within their complex environments. Continued efforts are now critical to integrate additional emerging optics technologies and deliver a first generation of environmental microscopes. The potential of this development to promote our understanding of the biology of this critical environment is enormous.

## Materials and Methods

### Fabrication of Chambers.

Plants and bacteria were grown in mesocosm chambers holding soil, water, and nutrients. Chambers consisted of glass slides (76 × 26 × 1 mm^3^, VWR) and polydimethylsiloxane (PDMS, SYLGARD 184, Sigma-Aldrich). PDMS with a 3 × 2 mm^2^ cross-section was used to seal the glass slides and for flexible supply of gas and fluids using syringes. Therefore, the chamber obtained had a volume of 4,290 mm^3^ (*SI Appendix*, Supplementary Text 1). Nutrients and index-matching liquid were infiltrated into the soil using two Ismatec Reglo peristaltic pumps (Cole-Parmer) when required. The fabricated mesocosm chambers were then mounted to the microscope using a custom-made sample holder (*SI Appendix*, Supplementary Text 4).

### Multispectral LSFM for Whole-Plant Environment Imaging.

The Gaussian beam from a four-channel laser source (488, 514, 561, and 633 nm, VersaLase, Laser 2000) was expanded to 2.6 mm in diameter (full width at half maximum) and split evenly into two illumination arms (*SI Appendix*, Supplementary Text 5). The homogeneous light sheet was generated using two Powell lenses (10° fan angle, LOCP-8.9R10-2.0, Laser Line Optics) and two cylindrical lenses (100 mm focal length, LJ1567RM-A, Thorlabs). The beam thickness has a full width half maximum of 50 µm, with a measured Rayleigh range of 1.7 mm. The image was projected through a 2× NA = 0.055 or 5× NA = 0.14 long working distance objective (Mitutoyo Plan Apo Infinity Corrected), a fluorescence emission filter changer (Four-Position Slider, ELL9, Thorlabs), and a tube lens (TTL200-A, Thorlabs) to a scientific camera (CMOS Camera, C11440-22CU, Hamamatsu). A three-axis translation stage was used for acquisition of large volumetric data. It consisted of two DC motor linear stages (M-VP-25XA, MKS) for horizontal displacement and a stepper motor linear stage (LNR50S/M, Thorlabs) for vertical displacement. Fluorescence and scattering signals were acquired serially. With a 2× objective, 7 × 60 × 35 mm^3^ can be imaged from image stacks of 200 slices (in step of 50 μm) at 10 vertical positions obtained in steps of 4 mm. Since the chambers were 3-mm thick, the total volume of the sample imaged was 3,600 mm^3^. Illumination using 633 nm was used to collect scattering signals generated by the plant. All other signals were used to collect fluorescence signals with a band pass (520 nm, 36 nm Edmund Optics) or a series of long pass (550, 600, and 650 nm, Thorlabs) emission filters. The chamber was attached to the three-axis stage by a custom-made holder. The holder was attached to a manual rotation stage (MSRP01/M, Thorlabs), and samples were positioned and translated along an axis forming an angle of ∼45° with the illumination and detection axes. The sample was placed in an acrylic tank filled with ∼10% sugar solution (refractive Index of 1.3478).

### Environmental Control.

Transparent soils are produced from granular substrates whose particles are made from transparent materials that have a refractive index approaching that of water (1.333). Nafion in the form of pellets was used to generate the transparent soil particles (4 × 3 mm, Ion Power, Inc.). The particles were fractured to a size similar to those found in sandy soils (0.25 to 1.25 mm) using a freezer mill (6850 Freezer/Mill, SPEX CertiPrep) and a series of sieves. pH and mineral ion concentration on the surface of the particles was then obtained by a series of chemical processes described earlier (32). Percoll (colloid suspension, GE Healthcare) was infiltrated into the soil to match the RI of the Nafion particles before imaging. Plants grew under the illumination generated by a light-emitting diode (LED) light panel composed of a red and blue light with 3:1 ratio and producing photosynthetic photon Flux of 240 µmol ⋅ s^−1^. The arrangement of the LED was designed to fit the sample holder and rotary stage. Water from the tank was circulated with miniature water pumps (480-122, RS Components) through a Peltier device (RS 693–7080, Components) with the temperature controlled using a TLK33 controller (Ascon Tecnologic). During the experiments, the temperature was set to 20 °C.

### Plant and Bacterial Culture.

Seeds of lettuce (*Lactuca sativa* all “year round,” Sutton Seeds) were surface sterilized by washing in 10% bleach for 15 min followed by thorough rinsing with sterile deionized water (DI H_2_O) before overnight germination on sterile distilled water agar plates. A single seedling (with ∼2 mm root length) was then transferred into an assembled, prefilled mesocosm containing transparent soil saturated in Murashige and Skoog Basal Medium (MS, Sigma-Aldrich) and stained with sulforrhodamineB (*SI Appendix*, Supplementary Text 1). *B. subtilis* NCIB 3610, GFP-labeled strain [NRS1473 (42)] was grown in MSgg medium (5 mM potassium phosphate and 100 mM MOPs adjusted to pH 7.0 and then supplemented with 2 mM MgCl_2_, 700 μM CaCl_2_, 50 μM MnCl_2_, 50 μM FeCl_3_, 1 μM ZnCl_2_, 2 μM thiamine, 0.5% glycerol [volume/volume], and 0.5% [weight/volume] glutamate) for 28 h at 18 °C, while shaking at 200 rpm. After incubation, the MSgg solution was replaced with MS to remove any carbon contained in the bacterial solution. Based on the optical density values (OD_600_), absorbance of the bacterial suspension in half-MS and the known correlation with colony-forming unit (CFU) for this strain, ∼2.0 × 10^6^ CFU were inoculated onto a sterile filter disk. The inoculated disk was then inserted into the mesocosm, just under the surface of the transparent soil, level with the plant seedling root. Mesocosms, with a lettuce seedling in each, were inoculated and subsequently incubated at 21 °C for 20 h. After this initial establishment period, the half-MS solution was removed and replaced with Percoll solution for index matching and imaging. Image capture was initiated in the morning and collected the following day. In total, six mesocosms inoculated with bacterial suspension and four individual mesocosms without inoculation of bacterial suspensions were studied with the system.

### Software Control.

The environmental microscope was operated through custom-made LabVIEW software (National Instrument). A single-board microcontroller (Arduino Mega 2560, RelChron Ltd.) controlled the laser output through RS232 external triggers. The growth light was powered by a DC power supply controlled by a USB-RLY08 relay (Devantech Limited).

### Image Acquisition and Processing.

Data from ∼2/3 of the microcosm was used for analysis. Processing was tailored to requirements for the quantification of root and particle geometry and quantification of bacterial cell density (*SI Appendix*, Supplementary Texts 6 and 7). Fluorescence and scattering signals were affine transformed with nearest-neighbor interpolation to correct for the angle of the scan (45°) used. Volume data were subsequently processed by the Lucy–Richardson deconvolution method with a light sheet point spread function (43). Overlapping regions were fused using a Laplace pyramid blend (44, 45). As soil is a textured material, it contains periodic (particles) that can be used to infer flat field corrections. The correction was based on a weight matrix computed from the mode value of the pixel intensity computed from neighboring pixels of an entire dataset and modification of the image intensity applied using Laplace pyramids method.

Image segmentation was used to extract the shape, structure, and spatial distribution of roots, bacteria, and soil particles (*SI Appendix*, Supplementary Text 7). The morphology of the root was obtained using a region-growing algorithm, and the resulting binary data were used to produce distance maps and to calculate the position of bacteria relative to the root surface (Movie S4). Only minor changes to the threshold values and position of the seeds were needed to account for variations in scattering intensity and movements of the root. Because sulforhodamine-B attaches only superficially to the soil particle, the signal was not sufficient to segregate the pore space from the core of the particle. Therefore, we overplayed the inverse of the GFP signal to improve the segmentation of the pore space, and a manual threshold followed by morphological operators (dilation and erosion) generated binary images of the pore space. The pore size was calculated using the local thickness metric (Movie S5 and *SI Appendix*, Supplementary Text 7).

Image processing methods were programmed using MATLAB using the Image Processing Toolbox (MathWorks). Segmentation and extraction of geometrical features were performed using MeVisLab (MeVis Medical Solutions AG). All software is freely available from https://github.com/LionelDupuy/SENSOIL.

### Calibration of Fluorescence Signal.

Dense bacterial suspensions were prepared in Percoll and measured by OD_600_. Suspensions prepared at 12 different OD_600_ in the range 1.2 × 10^−3^ to 3.0 were obtained by multiple dilutions. One milliliter of each bacterial suspension was transferred into mesocosm chambers, and stained soil particles were added to the chamber to adjust the focus of the environmental microscope. A full scan was acquired 2 mm above the particles (in steps of 50 µm and at two z levels 4 mm apart). Pixel intensity data were then correlated against OD_600_ values, and OD_600_ values were correlated to CFU counts. The estimation of bacterial cell density from image data were based on the combination of both correlations (*SI Appendix*, Supplementary Text 8). Calibration was made on suspensions and may therefore underestimate bacterial density in biofilms because of obscuration or changes in levels of expressions of the reporter gene.

### Indicators for Bacterial Activity.

Different indicators were used to map bacterial activity in the pore space. Bacterial cell density was estimated from the intensity of the green fluorescence signal. For all analyses, a pixel at a given time point is associated with three variables: pore size, distance from the root surface, and distance from the tip of the root. Pixels were subsequently classified into groups ($R_{k}$) related to their relative position along the root, to their position perpendicular to the root, or the size of the pore they are located in.

For each group of pixels $R_{k}$ at time *t*, the bacterial cell density D is defined as the estimated number of bacterial cell (CFU) in a unit soil volume expressed per unit volume of root (17), and the normalized density, D/〈D〉, was obtained (*SI Appendix*, Supplementary Text 8). The mobility of bacteria was quantified as the variance in bacterial density at a given location in soil. The first five time points were discarded from the computations to allow sufficient soil volume in the mature region of the root. Observation of the radial distribution of surrounding soil bacteria were made across the entire thickness of the soil from a diameter of 3 mm around the root center line. It was observed that bacteria in mature regions of the roots were distributed well within 1 mm from the root. Therefore, rhizosphere pixels were defined empirically as pixels, for which the distance from the root is less than 0.2 mm. Bulk soil pixels are defined as pixels which distance is more than 0.2 mm from the root surface. Pixels associated with the base of the root (mature part) are defined as pixels more than 2 mm away from the tip, at which point no elongation of cells was observed. Pixels associated with the base of the root are more than 2 mm away from the tip.

## Data Availability

The data in this study is available at https://doi.org/10.5281/zenodo.5650962.
